# The Attachment of Juvenile Mussels via Byssus Weakened by Contaminated Polyethylene Fibers

**DOI:** 10.3390/toxics12110768

**Published:** 2024-10-23

**Authors:** Wei Ma, Xuelian Wei, Fenglian Zeng, Ming Li, Ping Wang, Yingying Ye, Jiji Li

**Affiliations:** 1 Huaihe Basin Eco-Environment Monitoring and Scientific Research Center, Huaihe Basin Ecology and Environment Administration, MEE, Bengbu 233001, China; mawei2030@huaihejg.mee.gov.cn (W.M.); zengfenglian0220@163.com (F.Z.); kaifengliming@126.com (M.L.); wangping_0126@126.com (P.W.); 2 National Engineering Research Center for Marine Aquaculture, Zhejiang Ocean University, Zhoushan 316022, China; weixl1247@163.com (X.W.); yeyy@zjou.edu.cn (Y.Y.)

**Keywords:** juvenile mussels, ropes for attachment, polyethylene fibers, attachment behavior, heavy metals, PAHs

## Abstract

In the process of mussel farming, the rope for attachment is indispensable, as it provides a stable attachment environment for mussel seedlings, directly affecting their survival rate and growth quality. The objective of this study is to examine the contamination of ropes, composed of polyethylene fibers, by heavy metals and polycyclic aromatic hydrocarbons (PAHs) after three years of deployment and to assess its influence on the attachment and locomotion behaviors of juvenile mussels. Utilizing a laboratory simulation of the seedling wrapping process, a comparative analysis was conducted to evaluate the number of juvenile mussels attached and their movement distances when exposed to contaminated old ropes versus uncontaminated new ropes. The findings indicated that the old ropes markedly diminished the attachment rate of juvenile mussels and heightened their movement distances. In particular, juvenile mussels utilizing old ropes exhibited a final attachment rate of 15.0% and an average movement distance of 0.86 cm, whereas those using new ropes achieved a final attachment rate of 96.7% with an average movement distance of 0.26 cm. Further inspection found that heavy metals and PAHs were present in the old rope, among which the concentrations of Zn (17.127 μg/g) and Pb (22.905 μg/g) in heavy metals were high, and the concentrations of Phe (5.53 μg/kg), Fla (6.35 μg/kg), and Pyr (5.17 μg/kg) in PAHs exceeded the detection limits, which were the main source of pollution. This research underscores the potential risk that heavy metal and PAHs contamination pose to the health of juvenile mussels and the profitability of aquaculture, emphasizing the critical need for the regular replacement of clean ropes.

## 1. Introduction

Mussels play a crucial role in marine ecosystems, providing essential ecological roles and economic benefits. As natural “filters”, mussels efficiently sieve and consume suspended particles, organic debris, and microorganisms from seawater. This process not only effectively removes pollutants from the water, mitigating eutrophication, but also promotes the recycling of nutrients, thereby providing essential nutritional sources for other organisms within the marine ecosystem [1,2]. When mussels die, their shells remain. They become important parts of the marine ecosystem, helping to shape and protect it. These shells gradually accumulate on the seabed, forming shellfish reefs that alter the seabed’s topography and provide shelters, breeding grounds, and foraging areas for numerous marine species [3]. The existence of shellfish reefs fosters biodiversity, enhances ecosystem resilience and recovery, and is crucial for maintaining the overall stability of marine ecosystems [4]. Moreover, the emergence of mussel aquaculture has significantly contributed to sustainable seafood production [5,6].

Mussels are cultivated around the world, including in regions such as Europe, North America, and Asia, due to their high market demand and ecological benefits. In China, coastal regions like Zhoushan, Zhejiang, have seen rapid development in mussel aquaculture [7]. Cultivation techniques for these mussels are varied, primarily including floating raft suspension culture and bottom sowing for population enhancement [8,9]. These methods not only make optimal use of marine resources but also enable efficient, large-scale production of mussels. In recent years, with ongoing innovation and improvement in aquaculture technology, the farming scale of thick-shelled mussels *Mytilus coruscus* has expanded rapidly, especially in coastal regions such as Zhoushan, Zhejiang, China, where intensive and standardized farming models have emerged.

In the intricate realm of marine bivalve aquaculture management, the attachment phase of juvenile mussels signifies the inception of their growth cycle, thereby underlining its pivotal significance. Nonetheless, this phase is inherently susceptible to various potential threats to the safety of the mussel larvae, among which the contamination status of the ropes utilized for seedling attachment is a paramount concern from a biological standpoint, necessitating special attention and scrutiny. At present, the material used for mussel attachment rope is generally polyethylene fiber [10]. The ropes, acting as a bridge between juvenile shellfish and the aquaculture setting, are vulnerable to absorbing and building up pollutants from their surroundings after prolonged exposure to seawater. This accumulation can encompass persistent organic pollutants (POPs) and heavy metals, posing potential risks [11,12]. The POPs mainly include organochlorine pesticides (OCPs), polychlorinated biphenyl (PCBs), polycyclic aromatic hydrocarbon (PAHs), etc. Li et al. [13] studied 14 kinds of PAHs in the surface seawater off the coast of Zhejiang and showed that the content of Σ14PAHs ranges from 582.82~1208.30 ng/L in winter and 952.40~1201.70 ng/L in summer. Men et al. [14] showed that the PCBs content in the Daliao River Estuary was 5.51~40.28 ng/L. These pollutants may not only adhere directly to the surface of the seedling ropes but also infiltrate into their interiors, thereby coming into direct contact with juvenile shellfish during the seedling attachment process and posing a serious threat to their health. The POPs, such as organochlorine pesticides (OCPs), polychlorinated biphenyl (PCBs), polycyclic aromatic hydrocarbon (PAHs), etc., can compromise the survival rates and growth rates of juvenile shellfish by disrupting their physiological functions and genetic health [15,16]. Meanwhile, heavy metals like lead (Pb), cadmium (Cd) and mercury (Hg), due to their non-degradability and bioaccumulation properties, inflict long-term and profound damage on the nervous systems, kidneys, and bone development of juvenile shellfish [17,18]. Therefore, effectively controlling the pollution levels of ropes during the seedling attachment process to ensure a relatively clean and safe growth environment for juvenile shellfish is of great significance in enhancing aquaculture success rates, safeguarding the quality of shellfish products, and promoting the sustainable development of marine aquaculture.

This study simulated the seeding process in a laboratory setting to analyze the influence of contaminated ropes on the attachment of juvenile shellfish. The attachment capability of byssus was examined by quantifying the number of juvenile shellfish attached and measuring their movement distances. It is important to note that this study focused on the effects of toxic substances, such as heavy metals and PAHs, that accumulated on the used polyethylene ropes rather than on the physical aging of the ropes themselves, as previous studies and aquaculture practices have shown that juvenile mussels often prefer attaching to aged, rougher surfaces. Additionally, the contamination level of heavy metals and PAHs on the seeding ropes was detected to uncover the relationship between pollutants and the health status of juvenile shellfish, thereby providing scientific evidence and technical references for mussel aquaculture and marine ecological protection, with the aim of guiding production practices.

## 2. Materials and Methods

### 2.1. Sample Collection

**Figure 1:**
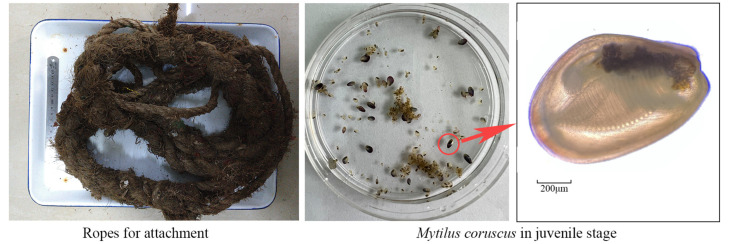
Experimental material.

The juvenile thick-shelled mussels and ropes used in the research were obtained from Shengsi Marine Science and Aquatic Products Research Institute in Shengsi, Zhoushan City, Zhejiang Province (Figure 1). The rope used in the test group underwent a three-year mussel farming cycle, while new ropes served as the control group.

### 2.2. Sample Processing

The retrieved juveniles were temporarily raised in clean artificial seawater in the laboratory to eliminate any potential contamination from the natural environment within their bodies. Before use, the artificial seawater was filtered through a 0.22 μm filter membrane to maintain an environment free of microplastics and other pollutants.

The ropes used in this study were all made of polyethylene fibers (diameter of 1 cm), with the same material composition for both the used and new ropes. The used ropes had been deployed as mussel attachment ropes in aquaculture sites for a period of three years, while the new ropes were unused and had not been exposed to any environmental conditions. Both the used and new ropes were cut into segments and disassembled to prepare samples for different experimental purposes. One portion of each rope type was allocated for heavy metal and PAHs measurements, while the other portion was used in the attachment experiments.

For the attachment experiments, the ropes were divided into thinner strands with a diameter of approximately 2 mm. Each strand was tied at both ends using corrosion-resistant metal wires coated with an anti-seawater varnish to prevent fraying and to ensure the integrity of the short strands during the experiments.

### 2.3. Experimental Treatments

After they had adapted, juveniles exhibiting uniform growth and size were selected for experimentation. These juveniles were divided into experimental and control groups, with each group comprising three replicate experiments and each replicate containing 20 juveniles. Glass slides measuring 7.8 cm in length and 2 cm in width were bisected longitudinally, with one half having a rope attached to its base, ensuring the rope was taut. The two halves were then reassembled with their 2 cm widths facing each other, forming an inverted triangular structure placed within a 6-well plate. The juveniles were positioned at the rope to mimic the seedling attachment process.

Maintaining all other conditions constantly, the experimental group utilized ropes that had been in use for three years, while the control group employed new ropes. The attachment status of the juveniles were observed every 24 h, with a record of the number of attached individuals. Additionally, the displacement distance of attached juvenile mussels from the rope and along the glass slide was measured.

### 2.4. Determination of Heavy Metal Concentration

Heavy metal concentrations in rope samples were determined using inductively coupled plasma–mass spectrometry (ICP-MS, NexION 300X, PerkinElmer, Waltham, MA, USA). The specific steps were as follows:

Approximately 0.5 g of homogenized rope sample was accurately weighed and placed in a Teflon digestion vessel. Then, 10 mL of a mixture of nitric acid and hydrochloric acid in a 3:1 ratio was added to the vessel. The samples were then digested using a microwave digestion system (MARS Xpress, CEM, Matthews, NC, USA) following a specific temperature and pressure program to ensure complete dissolution: The vessels were heated from room temperature to 120 °C over 3 min, then to 150 °C over 2 min, maintained at 150 °C for 6 min, then increased to 180 °C over 4 min, and maintained at 180 °C for 12 min. After digestion, the solutions were cooled to room temperature, filtered, and diluted to a final volume of 50 mL with deionized water.

Heavy metal analysis of digested samples was performed using the ICP-MS system (ICAP Q series) Calibration standards and quality control samples were prepared using certified reference materials to ensure accuracy and precision [19]. The ICP-MS system was calibrated with a series of standard solutions containing known target heavy metal concentrations. The concentration of Hg, Cu, Zn, As, Cd, Pb, and Cr in the sample was quantitatively determined by introducing the sample solution into ICP-MS. Quality control measures included blank, duplicate, and standard reference analysis for each batch of samples.

### 2.5. Determination of PAHs Concentration

PAHs concentrations in rope samples were determined using gas chromatography–mass spectrometry (GC-MS, TQ8050, Shimadzu Corporation, Kyoto, Japan).

Approximately 10 g of representative rope material was cut and thoroughly ground to a particle size less than 0.5 mm, ensuring efficient release of PAHs. Subsequently, an accelerated solvent extraction (ASE) process was utilized, employing a mixture of dichloromethane and n-hexane (1:1 vol/vol) as the extraction solvent. The extraction was performed at 100 °C and 1500 psi for three cycles, each lasting 10 min, to effectively extract the PAHs from the rope. The extract was then purified using a silica gel column to remove impurities and concentrated under a nitrogen stream to approximately 1 mL, at which point it was ready for GC-MS analysis.

The specific detection conditions for GC-MS are as follows. Chromatographic conditions: column type: SH-I-35Sil MS capillary column (30 m × 0.25 mm × 0.25 µm); temperature procedure: The initial temperature was maintained at 50 °C for 2 min, then heated up at 10 °C/min to 200 °C, and then heated up at 5 °C/min to 290 °C for 25 min. Inlet temperature: 300 °C; injection size: 1 µL, no shunt injection. Mass spectrum conditions: ion source temperature 230 °C, EI source, solvent delay time 3 min; data acquisition mode: SIM; detector voltage: tuned voltage + 0.2 kv, after 33 min, the absolute voltage value was 1.35 kv.

All samples were analyzed for the 16 US EPA priority PAHs, including fluoranthene (Fla), pyrene (Pyr), benzo[a]anthracene (BaA), chrysene (Chr), benzo[b]fluoranthene (BbF), benzo[k]fluoranthene (BkF), benzo[a]pyrene (BaP), indeno[1,2,3-cd]pyrene (IcdP), dibenzo[a,h]anthracene (DahA), benzo[g,h,i]perylene (BghiP), naphthalene (Nap), acenaphthylene (Acy), acenaphthene (Ace), fluorene (Flo), phenanthrene (Phe), and anthracene(Ant).

### 2.6. Data Analysis

All data are expressed as mean ± SD and were analyzed by one-way analysis of variance (ANOVA) using SPSS software (IBM SPSS statistics 26, New York, NY, USA). Differences were considered statistically significant at *p* < 0.05. The images were drawn with Origin 2022.

## 3. Results and Discussion

In marine shellfish aquaculture, the settlement and mobility behaviors of juvenile are directly related to their growth and development as well as the ultimate economic benefits of aquaculture. Environmental factors such as deteriorating water quality (such as heavy metal and PAHs pollution), unsuitable substrate, inadequate lighting, or temperature fluctuations can all potentially affect the adhesion ability of juveniles [20,21,22]. Furthermore, the health status, nutritional condition, and genetic background of the juvenile mussels themselves may also influence their environmental adaptability and settlement ability [23,24].

In order to reveal the effects of environmental pollutants on the behavior of juveniles, we simulated the process of seedling wrapping and compared the attachment and distance of juvenile mussels on the glass sheet under the conditions of using contaminated polyethylene fiber ropes (experimental group) and uncontaminated new ropes (control group). The following is an in-depth discussion of the experimental results.

### 3.1. Determination and Pollution Analysis of Heavy Metals and PAHs in Polyethylene Fiber

#### 3.1.1. Heavy Metal

After testing, we detected seven kinds of heavy metals from the ropes (Table S1). Their concentrations in descending order were Hg (0.011 μg/g), Cr (0.013 μg/g), Cd (0.045 μg/g), As (2.265 μg/g), Cu (9.232 μg/g), Zn (17.127 μg/g), and Pb (22.905 μg/g) in the used ropes. This shows that the seedling rope is gradually polluted by heavy metals after long-term use. Pb showed a mean of 22.905 μg/g, indicating a significant contamination source for Pb levels in mussels. Among them, the concentrations of Hg, Cr, and Cd were very low, indicating that the pollution level of these three heavy metals on the seedling rope was still relatively low. The relatively high concentrations of As and Cu suggest that the concentrations of these two heavy metals in the aquaculture area reached a certain level. Meanwhile, the concentrations of Zn and Pb were as high as 17.127 μg/g and 22.905 μg/g, respectively, indicating that these two heavy metals have become significant pollution sources in the aquaculture area. In contrast, none of these heavy metals were detected in the new ropes (control), as their concentrations were all below the detection limits.

#### 3.1.2. PAHs

We tested the content of PAHs in the rope, and a total of 16 PAHs were selected. As shown in Table S2, the test results showed that only three PAHs were detected, among which the content of phenanthrene (Phe) was 5.53 μg/kg, fluoranthene (Fla) was 6.35 μg/kg, and pyrene (Pyr) was 5.17 μg/kg. The remaining polyaromatic hydrocarbons were not detected. The test showed that the polyethylene fiber rope used for a long time was contaminated by PAHs, mainly by Phe, Fla, and Pyr. Similar to the heavy metals, the concentrations of PAHs in the new ropes (control) were all below the detection limits.

#### 3.1.3. Pollution Analysis

Zhoushan City is an important gathering place of aquatic processing enterprises in China, and the harbor industry has developed rapidly. The survey shows that aquatic product processing in Zhoushan City with high water consumption mainly includes four categories: tuna processing, squid processing, surimi processing, and chitin utilization. The survey shows that tuna processing enterprises need to discharge about 17 t/t of waste water to produce a unit of product, squid processing enterprises need to discharge about 25 t/t of waste water to produce a unit of product, squid processing enterprises need to discharge about 25 t/t of waste water to produce a unit of product, and chitin utilization enterprises need to discharge up to 150 t/t of waste water to produce a unit of product [25]. The marine environment of this area has been seriously affected by the discharge of industrial sewage and urban domestic sewage, which leads to a great amount of heavy metals, PAHs, and other pollutants in seawater. Heavy metals and PAHs are deposited on the surface of the rope through water circulation and gradually penetrate, causing damage to the material of the rope.

The transport of contaminants such as heavy metals and PAHs deposited on the rope from the plastic filament to the mussels is a complex process. This involves several steps, including the desorption of contaminants from the plastic surface, transport through the water column, and absorption by the mussels [26]. At present, the mechanism of transport of contaminants from microplastics to mussels is still not fully understood. A number of studies have investigated the transfer of contaminants from microplastics to mussels. For example, Li et al. [26] studied the effects of PAH-contaminated microplastic filaments on thick-shell mussel larvae and found that surface contact is the main route of pollutant transfer to thick-shell mussel larvae. The results of this study showed that the attachment of juveniles to the rope was also the reason for the transfer of pollutants such as heavy metals into mussels. The larval stage of the mussel mainly refers to the trochal larva and D-type larva after the fertilized egg hatches, and the larva in this stage has not yet been shaped and is in the early stage of growth and development. The juvenile stage includes to the series of development processes leading to metamorphosis and the camp attachment life of the larvae. Therefore, juveniles are attached to the rope during seedling encapsulation, and heavy metals are highly likely to be transferred from the rope to the juvenile and gradually accumulate with the growth and development of the mussel, affecting the growth rate and quality of the mussel and posing a serious threat to the mussel culture industry. More crucially, when humans consume these contaminated mussels, the heavy metals and PAHs enter the human body via the food chain. Long-term accumulation of these metals can lead to neurological impairments, abnormal liver and kidney functions, and even the development of cancer, posing serious health risks [27,28].

### 3.2. The Impact of Contaminated Polyethylene Fibers on the Attachment Quantity of Juveniles

Table 1 presents the daily average attachment status and quantity of juvenile mussels in both the experimental and control groups. In the experimental group, on the first day, an average of 12 juveniles remained unattached, while the remaining 8 were evenly distributed, with half attached to the ropes and half to the glass slides. Subsequently, the number of unattached juveniles gradually increased, and the number of attached ones decreased until day 7, when the attachment status stabilized with approximately 17 juveniles remaining unattached, 1 attached to the ropes, and 1 attached to the glass slide. In contrast, the control group exhibited a different trend. On the first day, nearly all juveniles attached, with approximately 13 attaching to the ropes, 5 to the glass slides, and only 2 remaining unattached. Over time, the number of attached juveniles gradually increased, stabilizing by day 6 with 1 unattached, 17 attached to the ropes, and 2 attached to the glass slides.

**Figure 2:**
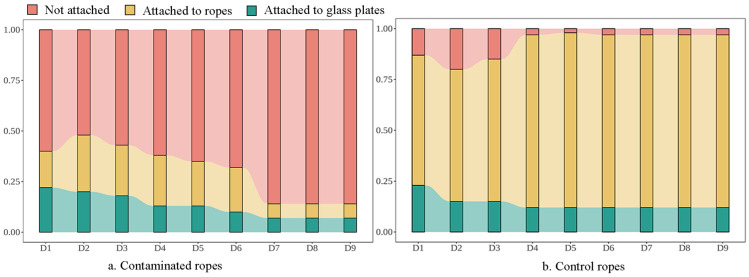
The proportion of attachment number of juvenile mussels.

Presenting the attachment status of juvenile mussels through images (Figure 2) allows for a more intuitive observation of the gradual decrease in the attachment ratio of juveniles in the experimental group over time. The final stable attachment rate of mussels onto the old ropes was 16%, with half of them attached to the ropes and the other half to the glass plates. In contrast, the attachment ratio of juveniles in the control group gradually increased, reaching a stable attachment rate of 96.7%, and the majority of these juveniles preferred to attach to the ropes (85%).

It is worth noting that physical changes in ropes, such as increased surface roughness and biofilm formation, could theoretically enhance the attachment of juvenile mussels. However, despite these favorable physical properties in the contaminated ropes, the attachment rate in this group was significantly lower than in the control group, where the ropes were new and smoother. This suggests that the negative effects of chemical contaminants outweighed any positive influence of physical changes, indicating that chemical contamination played a dominant role in reducing the attachment rate observed in the contaminated ropes group.

Firstly, the rope is made of polyethylene fiber, which inevitably undergoes degradation due to environmental factors during prolonged usage, such as salt in seawater, microbial and algal attachment, and UV radiation. These factors alter the surface properties of the ropes. Contaminated rope surfaces may accumulate substantial biofilms, organic pollutants, and inorganic sediments, modifying their roughness and hydrophilicity while potentially releasing harmful chemicals (e.g., heavy metals, PAHs, and degradation products of plastic additives) to juveniles [29]. These changes can adversely affect juvenile shellfish settlement behaviors, as juveniles preferentially select rough, microbe-rich, and chemically suitable surfaces for attachment [10].

Secondly, from an ecological perspective, the preference of juvenile shellfish for different attachment bases, such as ropes and glass plate, might be related to their natural habits and habitat requirements. In the control group, juveniles were more likely to attach to the rope, which may provide an environment (such as roughness and microbial community) closer to natural substrate, which is conducive to the fixation and growth of juveniles. Lao et al. [30] conducted a study on the survival of *Pinctada margaritifera* with different attachment bases, and the experimental results showed that the survival density of nymphals attached to ropes was the highest, which was consistent with the results of this study. The decrease in the attachment rate of juvenile mussels in the experimental group may reflect the negative effect of contaminated seedling rope on the living environment of juveniles. Pollutants may directly impair juvenile physiology, affecting their ability to secrete attachment proteins or disrupting signaling pathways involved in substrate recognition and selection [24,31,32]. Furthermore, pollution may alter the microbial community structure on rope surfaces, influencing the crucial juvenile shellfish–microbe interactions essential for juvenile settlement and growth [33].

From a behavioral biology standpoint, juvenile settlement is a complex adaptive response regulated by various internal and external factors. The reduced settlement ratio in the experimental group may represent an adaptive adjustment by juveniles in response to an unfavorable environment. Juveniles may assess substrate quality by sensing chemical signals, physical structures, or microbial community changes on rope surfaces, thereby making decisions regarding attachment or migration [34]. The steady increase in settlement rate in the control group indicates that new, uncontaminated ropes provide a suitable attachment environment conducive to juvenile growth and development.

### 3.3. The Impact of Contaminated Polyethylene Fibers on the Movement Distance of Juveniles

**Figure 3:**
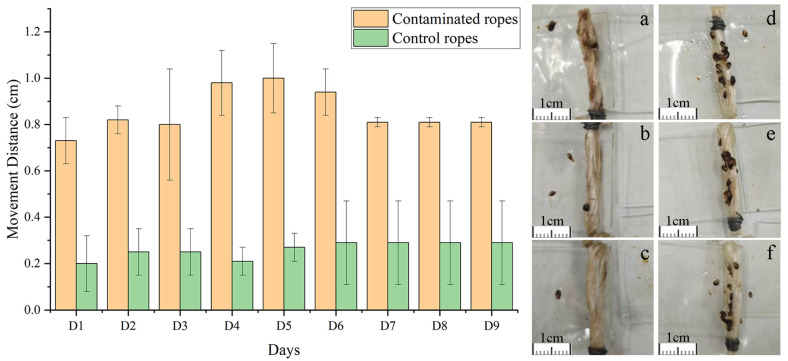
Statistics on the movement distance of juvenile mussels. In the figure, (**a**–**c**) represent the contaminated ropes, while (**d**–**f**) represent the control ropes.

Analysis of the migration distances of juvenile mussels attached to glass plates revealed that within the experimental group, the average migration distance on the first day was 0.74 cm. Subsequently, this distance progressively increased, peaking at 1.00 cm on the fifth day. Following this peak, the migration distance gradually decreased, with an average of 0.81 cm recorded during the final day of measurement. In contrast, the control group exhibited no significant fluctuations in their average migration distance over the course of nine days (Figure 3). A statistical summary of the average migration distances for both the experimental and control groups over nine days is presented in Table 2. Specifically, the experimental group exhibited an average migration distance of 0.86 cm, while the control group demonstrated an average of only 0.26 cm. These data indicate that the average migration distance of the experimental group was significantly higher than that of the control group (Table 2).

In the experimental group, juvenile mussels exhibited mobility from day one, with an average movement distance of 0.74 cm. Over time, the movement distance gradually increased, peaking at 1.00 cm on the fifth day. This trend suggests that the juveniles in the experimental group initially attempted to find more suitable attachment sites or escape the adverse environment by increasing their mobility due to discomfort with the contaminated rope environment. However, with extended exposure, the juveniles might have gradually adapted to the polluted conditions or experienced reduced mobility due to energy depletion and physiological stress, resulting in a decrease in movement distance after the fifth day, although remaining above the initial level. At the final measurement, the average movement distance was 0.81 cm, indicating some residual adaptability. In sharp contrast, the juvenile shellfish in the control group maintained a relatively stable movement distance throughout the experimental period, averaging 0.26 cm. This demonstrates that under uncontaminated rope conditions, juvenile shellfish maintain a stable behavioral pattern without the need for frequent movements to search for more suitable attachment environments. This result further confirms that environmental pollutants significantly influence juvenile behavioral patterns.

Although the contaminated ropes might have had more favorable physical characteristics for mussel attachment, such as increased surface roughness, the juvenile mussels in the experimental group exhibited significantly greater movement distances compared to the control group. This behavior suggests an avoidance response to the contaminated ropes, further indicating that chemical contamination, rather than physical changes, was the primary factor affecting mussel movement in this study.

The increased movement distance of the juveniles in the experimental group may be related to their stress response to the polluted environment. Pollutants can adversely affect juvenile mussels through various pathways, including altering water quality, changing rope surface properties, and disrupting physiological functions. For instance, pollutants may exert toxic effects on the juvenile nervous or motor systems, impairing their mobility. Additionally, pollutants can affect energy metabolism and nutrient absorption, further weakening mobility [35,36]. Lastly, pollutants may reduce dissolved oxygen levels in the water, affecting juvenile respiration, or alter the roughness and chemical composition of the rope surface, compromising juvenile shellfish attachment stability [37,38]. These adverse factors compel juveniles to increase their movements in response to environmental stress. Nevertheless, over time, juveniles may mitigate the negative effects of pollutants through physiological adjustments and behavioral adaptations. For example, they might adjust metabolic pathways to conserve energy or alter movement strategies to reduce exposure to pollutants [39,40]. Such adaptive adjustments enable juvenile mussels to maintain some level of activity, albeit with reduced movement distances later on.

## 4. Conclusions

Through the experiment, we found that the rope to which juvenile mussels attach was polluted by heavy metals and PAHs, among which the heavy metals were mainly Zn and Pb, and the polycyclic aromatic hydrocarbons were mainly Phe, Fla, and Pyr. In the mussel wrap experiment, we found that the contaminated rope affected the attachment and movement behavior of juveniles, which led to a decrease in the attachment rate of juveniles and an increase in the moving distance. Long-term use of unreplaced rope will make the rope accumulate more and more harmful substances (such as heavy metals and PAHs), and there is a great possibility of transfer to the juvenile mussels. Although physical changes in the ropes (e.g., surface roughness and biofilm formation) might influence attachment, our findings indicate that chemical contamination was the primary factor affecting the behavior of juvenile mussels. Therefore, in order to ensure the healthy growth of juveniles, the rope should be replaced regularly with clean and uncontaminated rope so as to improve the attachment rate and survival rate of juvenile mussels and promote the sustainable development of shellfish aquaculture.

## Figures and Tables

**Table 1:** Statistical table of the average attachment quantity of juvenile mussels.

Days	Number of Attachments					
	Not Attached		Attached to Ropes		Attached to Glass Plates	
	Contaminated Ropes	Control Ropes	Contaminated Ropes	Control Ropes	Contaminated Ropes	Control Ropes
D1	12.00 ± 2.65	2.67 ± 2.89	3.67 ± 1.15	12.66 ± 2.08	4.33 ± 1.53	4.67 ± 2.08
D2	10.33± 3.51	4.00 ± 3.61	5.67 ± 1.53	13.00 ± 1.73	4.00 ± 2.00	3.00 ± 2.65
D3	11.33 ± 2.08	3.00 ± 2.65	5.00 ± 1.00	14.00 ± 1.00	3.67 ± 2.89	3.00 ± 2.65
D4	12.33 ± 3.21	0.67 ± 1.15	5.00 ± 3.00	17.00 ± 0.00	2.67 ± 2.08	2.33 ± 1.15
D5	13.00 ± 3.46	0.33 ± 0.58	4.33 ± 3.51	17.34 ± 0.58	2.67 ± 2.08	2.33 ± 1.15
D6	13.67 ± 3.06	0.67 ± 1.15	4.33 ± 3.51	17.00 ± 1.00	2.00 ± 1.00	2.33 ± 1.53
D7	17.33 ± 1.53	0.67 ± 1.15	1.33 ± 1.15	17.00 ± 1.00	1.33 ± 0.58	2.33 ± 1.53
D8	17.33 ± 1.53	0.67 ± 1.15	1.33 ± 1.15	17.00 ± 1.00	1.33 ± 0.58	2.33 ± 1.53
D9	17.33 ± 1.53	0.67 ± 1.15	1.33 ± 1.15	17.00 ± 1.00	1.33 ± 0.58	2.33 ± 1.53

**Table 2:** Average movement distance by juvenile mussels attached to glass plates over a 9-day period.

Days	Movement Distance (cm)	
	Experimental Group	Control Group
D1	0.73 ± 0.10	0.20 ± 0.12
D2	0.82 ± 0.06	0.25 ± 0.10
D3	0.80 ± 0.24	0.25 ± 0.10
D4	0.98 ± 0.14	0.21 ± 0.06
D5	1.00 ± 0.15	0.23 ± 0.06
D6	0.94 ± 0.10	0.29 ± 0.18
D7	0.81 ± 0.02	0.29 ± 0.18
D8	0.81 ± 0.02	0.29 ± 0.18
D9	0.81 ± 0.02	0.29 ± 0.18
Mean Movement Distance	0.86 ± 0.09 *	0.26 ± 0.04

Notes: *: *p* < 0.05.

## Data Availability

Data will be provided by the corresponding author upon reasonable request.

## Supplementary Materials

The following supporting information can be downloaded at https://www.mdpi.com/article/10.3390/toxics12110768/s1, Table S1: Test results of heavy metal in ropes; Table S2: Test result of PAHs in ropes.
