# Supplementary material for: The Attachment of Juvenile Mussels via Byssus Weakened by Contaminated Polyethylene Fibers
Source: Toxics. 2024 Oct 23;12(11):768. doi: 10.3390/toxics12110768 (PMC11598145; doi:10.3390/toxics12110768)
Supplement: Supplementary file 1 [file toxics-12-00768-s001.zip › toxics-3242000-supplementary.pdf]

Table S1. Test results of heavy metal in ropes.

Number	Heavy Metal	Concentration (μg/g)		Detection limit (μg/g)
		contaminated ropes	control ropes	
1	Hg	0.011±0.009	-	0.002
2	Cr	0.013±0.008	-	0.022
3	Cd	0.045±0.043	-	0.009
4	As	2.265±1.230	-	0.024
5	Cu	9.232±4.366	-	0.015
6	Zn	17.127±6.429	-	0.132
7	Pb	22.905±13.302	-	0.018

Note: - means under detection limit.

Table S2. Test result of PAHs in ropes.

Number	PAHs	Abbreviation	Concentration (μg/Kg)		Detection limit (μg/Kg)
			contaminated ropes	control ropes	
1	Naphthalene	Nap	-	-	1.0
2	Acenaphthylene	Acy	-	-	0.6
3	Acenaphthene	Ace	-	-	0.6
4	Fluorene	Flo	-	-	0.8
5	Phenanthrene	Phe	5.53±0.12	-	1.2
6	Anthracene	Ant	-	-	0.6
7	Fluoranthene	Fla	6.35±0.37	-	0.6
8	Pyrene	Pyr	5.17±0.49	-	1.2
9	Benzo[a]anthracene	BaA	-	-	0.6
10	Chrysene	Chr	-	-	0.6
11	Benzo[b]fluoranthene	BbF	-	-	0.6
12	Benzo[k]fluoranthene	BkF	-	-	0.6
13	Benzo[a]pyrene	BaP	-	-	0.6
14	Indeno[1,2,3-cd]pyrene	IcdP	-	-	0.8
15	Dibenzo[a,h]anthracene	DahA	-	-	0.8
16	Benzo[ghi]perylene	BghiP	-	-	0.8

Note: - means under detection limit.
